# Molecular Epidemiological Investigation and Management of Outbreak Caused by Carbapenem-Resistant *Acinetobacter baumannii* in a Neonatal Intensive Care Unit

**DOI:** 10.3390/microorganisms11041073

**Published:** 2023-04-20

**Authors:** Jia Jie Woon, Azanna Ahmad Kamar, Cindy Shuan Ju Teh, Nuryana Idris, Rosliza Zhazali, Suzana Saaibon, Harvinderjit Kaur Basauhra Singh, Jasreena Kaur Gill Charanjeet Singh, Adeeba Kamarulzaman, Sasheela Ponnampalavanar

**Affiliations:** 1 Department of Medical Microbiology, Faculty of Medicine, University of Malaya, Wilayah Persekutuan Kuala Lumpur 50603, Malaysia; jiajiewoon93@hotmail.com (J.J.W.); nuryana@ummc.edu.my (N.I.); 2 Department of Paediatrics, Faculty of Medicine, University of Malaya, Wilayah Persekutuan Kuala Lumpur 50603, Malaysia; azanna@um.edu.my; 3 Department of Infection Control, University of Malaya Medical Centre, Wilayah Persekutuan Kuala Lumpur 50603, Malaysia; roslizazh@ummc.edu.my (R.Z.); suzanas@ummc.edu.my (S.S.); 4 Department of Nursing, University of Malaya Medical Centre, Wilayah Persekutuan Kuala Lumpur 50603, Malaysia; harvinderjit@ummc.edu.my; 5 Department of Pharmacy, University of Malaya Medical Centre, Wilayah Persekutuan Kuala Lumpur 50603, Malaysia; jasreena@ummc.edu.my; 6 Department of Medicine, Faculty of Medicine, University of Malaya, Wilayah Persekutuan Kuala Lumpur 50603, Malaysia; adeeba@um.edu.my

**Keywords:** molecular epidemiology, carbapenem-resistant *Acinetobacter baumannii*, outbreak, neonatal intensive care unit

## Abstract

The present study describes an epidemiological investigation into a carbapenem-resistant *Acinetobacter baumannii* (CRAB) outbreak, which had occurred in a neonatal intensive care unit (NICU), and the subsequent strengthening of infection control interventions. Upon the onset of the outbreak, existing infection control interventions were reviewed, and a set of containment measures were instituted. All CRAB isolates were characterized in terms of antimicrobial susceptibility testing and their genetic relatedness. The investigation process identified gaps within the NICU’s existing infection control measures, which had likely resulted in the outbreak. CRAB was isolated from nine preterm infants: five colonized and four infected. All five colonized patients were discharged well. However, three out of four of the infected infants died. Outbreak investigation and genomic subtyping of environmental swabs revealed that mini syringe drivers shared between patients and a sink in the milk preparation room had served as CRAB reservoirs with possible transmission via the hands of healthcare workers. Implementation of immediate actions such as reinforcement of hand hygiene practices, intensified environmental cleaning, geographical cohorting, reviewing of milk handling practices and sink management protocol had resulted in no further CRAB isolation. The CRAB outbreak in the NICU underlines the importance of consistent compliance with infection-control interventions. Integration of epidemiological and microbiological data, together with comprehensive preventive measures, successfully brought the outbreak to a halt.

## 1. Introduction

Carbapenem-resistant *Acinetobacter baumannii* (CRAB) is regarded as one of the main culprits for nosocomial infections and outbreaks [1,2,3]. Its recent emergence and increasing prevalence in intensive care units (ICUs) has gained notoriety in clinical settings as it poses serious threats to debilitated patients, causing infections that are normally associated with significant morbidity and mortality [4].

Preterm infants admitted to NICUs are vulnerable to healthcare-associated infections [5]. Factors such as low birth weight, prematurity, and immature innate immunity act as the causal or contributory factors for CRAB acquisition and infection [6]. Various serious infections can be caused by CRAB, including meningitis, pneumonia, necrotizing enterocolitis and bacteremia, thus putting neonates at the risk of prolonged hospital stays, high rates of re-admission and in-hospital mortality [7].

To date, CRAB outbreaks in NICUs are increasingly reported worldwide, mostly caused by strains isolated from exogenous sources, including medical devices such as vascular catheters and ventilator equipment as well as frequently touched surfaces around the patients such as sinks, tables, and beds [8,9,10]. Additionally, outbreaks due to cross-transmission between patients and healthcare workers are also reported in the literature [11]. 

The present study describes the epidemiological investigation of an outbreak caused by CRAB in a NICU as well as the subsequent strengthening of a series of infection control measures that led to successful containment of the outbreak.

## 2. Materials and Methods

### 2.1. Ethical Clearance

Medical ethics in conducting research on clinical isolates and accessing the patients’ clinical data were approved by the UMMC Medical Ethics Committee (MEC no. 1073.21). 

### 2.2. Study Design and Study Population

This is a retrospective descriptive study of an outbreak that occurred in the University of Malaya Medical Centre (UMMC). The study was conducted in the 27-bedded level III tertiary NICU, consisting of 3 isolation rooms and 2 open spaces. The nurse-to-patient ratio was 2:1. The studied sample included all infants with positive cultures of CRAB and admitted into NICU from 25 December 2016 to 8 February 2017. 

### 2.3. Outbreak Investigation

In January 2017, the hospital multidrug-resistant organisms (MDROs) surveillance system picked up an increase in CRAB in the NICU. A discernible increase in CRAB cases from a baseline rate of 0–1 case/month in preceding years to five cases prompted the declaration of an outbreak. 

As soon as the first case of CRAB was identified, a multidisciplinary outbreak management team comprising the NICU doctors, matrons, ward managers, pharmacist, ward infection control link nurses, infection control team and the cleaning staff was formed promptly to achieve effective outbreak containment. The hospital management and all relevant stakeholders were notified to ensure that the investigation was carried out without any obstruction and to ensure support in terms of additional supplies and staff required to manage the outbreak. Various infection control and prevention measures were rapidly initiated to avoid further acquisition and spread of the outbreak. This included cohorting of patients, review and observation of standing infection control practices in the NICU and decontamination of environments, which will be described in detail below. 

#### 2.3.1. Case Definition

A case was defined as any patient admitted into the NICU from January 2017 onward who had CRAB isolated from either clinical or screening samples more than 48 h after admission or more than 24 h after instrumentation in the NICU. A colonized infant was defined as any patient admitted into the NICU who had CRAB isolated from non-sterile sites such as skin, rectum or pharynx without signs of infection.

#### 2.3.2. Active Case Finding and Surveillance

Enhanced passive surveillance and screening of close contacts were initiated to determine the extent of the outbreak. Seven close contacts were identified. A close contact was defined as infants who were in adjacent bassinets and/or shared the same healthcare worker. Infants’ demographics, epidemiological and clinical data including gestational age, admission diagnosis, risk factors, procedures, devices in situ, comorbidities and 30-days mortality were retrieved from patients’ medical records. The movements of patients and healthcare personnel within the NICU were also studied. Details of the infants’ mothers’ previous or current infection or colonized status as well as practices related to handling of expressed breast milk (EBM) were also obtained. 

A line list containing the key information of each case, which included identifying information, demographic, clinical and risk factors, was created. The risk factors were identified based on the known understanding of the transmission characteristics of *Acinetobacter baumannii* and by searching for similar outbreaks in the literature, which is available in the Worldwide Database for Nosocomial Outbreaks. Information regarding risk factors such as the presence of invasive devices, equipment, medication and having underwent any procedures was collected. The data were analyzed, and a descriptive epidemiology focusing on time, place and person was prepared to identify the common characteristics and sources to assist in further investigation as well as to initiate infection-prevention measures. An epidemiology curve was plotted to visualize the distribution of cases over time, its relationship to the endemic cases and the magnitude of the outbreak. 

#### 2.3.3. Review of Existing Infection Control Practices (ICP) and Further Strengthening of the Practices 

A review of infection-prevention protocols and direct observation of patient care practices in the NICU, including central line-associated bloodstream infection (CLABSI) prevention bundle, VAP prevention bundle, milk handling and storage protocol, equipment disinfection and hand hygiene compliance, were conducted by the infection control nurse and the NICU managers. The findings were immediately communicated to all NICU staff. Based on the observation of practices, selected environmental swabs were obtained to assist in determining the possible source(s) of the CRAB. Swabs from 26 environmental/equipment surfaces along with 10 healthcare workers’ fingerprints were obtained. Based on observation of infection-prevention practices and following discussions, a multifaceted infection control strategy was instituted concomitantly with the outbreak investigation.

#### 2.3.4. Infection Control Intervention

Upon identification and isolation of the first CRAB, all existing infection control measures were strengthened to prevent further transmission. Infants with positive CRAB cultures were cohorted into isolation rooms, which were geographically separated from other infants. Contact precautions were practiced by all healthcare workers (HCWs). All cleaning staff were retrained regarding the appropriate cleaning protocols, and their practices, compliance and competencies were assessed by cleaning audits after each decontamination. HCWs were re-educated on proper donning and doffing of personal protective equipment gear, compliance with care bundles and on their hand-hygiene practice. Environmental decontamination was conducted vigorously in NICU environments using sodium hypochlorite 1000 ppm (Clorox^®^, The Clorox Company, Oakland, CA, USA) solution. Cleaning and disinfection of equipment, especially shared equipment, took place. 

#### 2.3.5. Strain Identification and Antimicrobial Susceptibility Testing

CRAB identity was confirmed via a duplex polymerase chain reaction [12]. Their respective antimicrobial susceptibility patterns were determined using the Kirby–Bauer disk diffusion method. The susceptibility of *A. baumannii* isolates to colistin was determined using broth the microdilution method according to Clinical and Laboratory Standard Institute (CLSI) guidelines. 

#### 2.3.6. Molecular Sub-Typing by Pulsed-Field Gel Electrophoresis (PFGE)

Genetic relatedness of clinical and environmental isolates was revealed via PFGE using *Apa*I (Promega, Madison, WI, USA) as the restriction enzymes [13]. The restriction fragments were separated using a BioRad CHEF Mapper electrophoresis system, with a running time of 20 h at 14 °C, using a pulse ramp rate changing from 2 s to 40 s. The resulting PFGE patterns were analyzed using BioNumerics version 6.0 software (Applied Maths, Kortrijk, Belgium).

## 3. Results

### 3.1. Outbreak Description and Patients’ Clinical Characteristics

**Figure 1:**
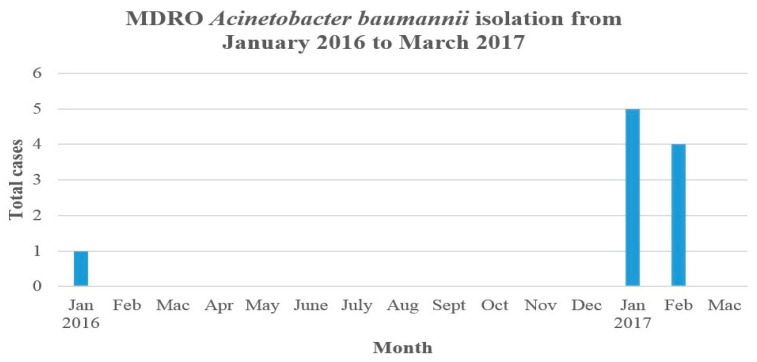
Isolation of multidrug-resistant *Acinetobacter baumannii* in the NICU from January 2016 to March 2017.

Prior to December 2016, the number of annual infections and colonizations caused by CRAB in the NICU over the previous five years ranged from zero to one case per year (Figure 1). From January to February 2017, a spike in the isolation of CRAB was reported among neonates admitted to the NICU. A total of nine preterm infants (five in January and four in February) were found to have CRAB isolated from various clinical and screening specimens. A line listing of the cases is summarized in Table 1. Among the nine infants, four infants showed signs of infection, whereas two showed evidence of bacteremia, while the other two developed pneumonia. Three deaths were reported (two with bacteremia and one with pneumonia). The other five infants were colonized with CRAB, which were isolated from rectal, trachea and eyes. They were discharged well. The mean gestational age, body weight and age at which CRAB was isolated was 30.7± SD 2.49 weeks, 1347± SD 403 g and 16± SD 8.81 days of life, respectively. 

**Figure 2:**
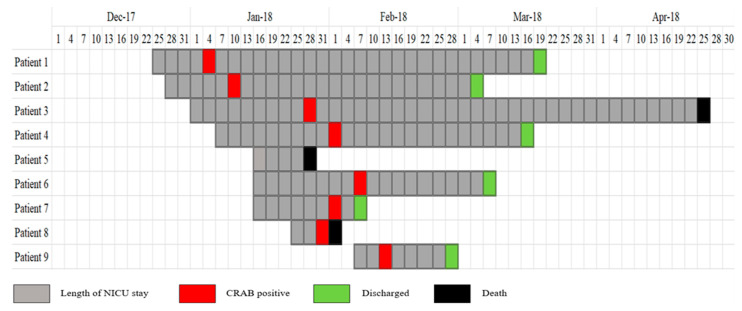
Temporal relationship between 9 infants with carbapenem-resistant *Acinetobacter baumannii* colonization or infection identified throughout the outbreak.

The index case was identified on the 3rd of January when CRAB was isolated from an infant’s tracheal aspirate (Patient 1) at the age of 10 days after birth. Subsequently, this pathogen was isolated from eight other infants within 40 days, with the second case identified on the 10th of January. The timeline of the outbreak occurrence is illustrated in Figure 2.

### 3.2. Microbiological and Molecular Subtyping Result

Nine clinical and four environmental isolates of *A. baumannii* were obtained, and the details of environmental culture surveillance are summarized in Table 2. All the clinical isolates of *A. baumannii* were found to be resistant to all classes of antibiotics, except to colistin. The minimum inhibitory concentration of colistin towards CRAB was found to be 1 µg/mL. Environmental culture surveillance and antimicrobial susceptibility testing revealed that isolates from one mini syringe driver, one sink in the milk preparation room and the fingerprints of an HCW (taken prior to handwashing and after touching an infected patient) shared similar antibiograms with those of clinical isolates. The antimicrobial susceptibility pattern of the isolates is summarized in Table 3.

**Figure 3:**
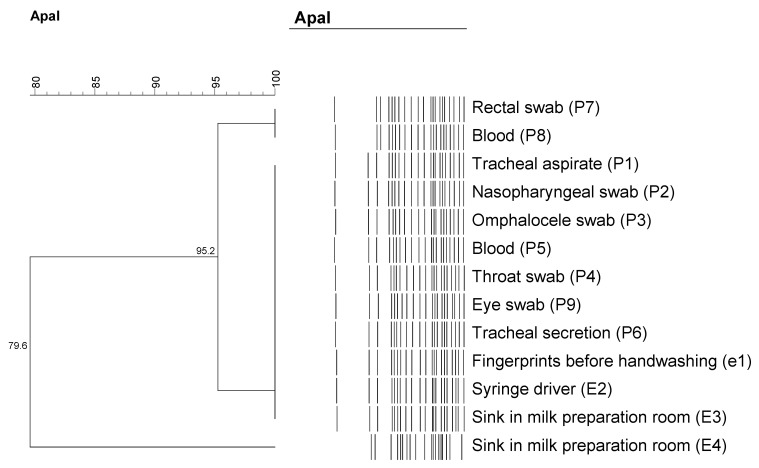
Dendrogram plot: result of molecular typing of CRAB from different sources and their percentage of similarity.

PFGE analysis subtyped these isolates into 3 pulsotypes with one predominant cluster, which accounted for 12 out of 13 isolates. All isolates in this cluster were grouped together with similarity of more than 90%, suggesting that the outbreak was caused by a single clone. Identical banding patterns shared between clinical and environmental isolates in this cluster provided the evidence that the mini-syringe driver and sink from the milk preparation room with the possible vector being the hands of HCW may have possibly served as potential exogenous sources of CRAB. The dendrogram generated from this PFGE analysis is shown in Figure 3.

### 3.3. Outcomes of Evaluation of Current ICP

Gaps in ICP practices were identified. ICP practices were not found to be standardized and HCWs were also not familiar with existing protocols. These included equipment- and environment-cleaning practices as well as the handling of breast milk. There was no proper milk-handling and storage protocol available. The milk-handling process was not conducted in a consistent manner, and there was a breach in the equipment cleaning procedure. The EBM was not stored immediately after receiving it from mothers. Furthermore, education on how to handle the expressed milk covering self-hygiene, cleaning of milk pumps, storage and transport of expressed breast milk was not consistently given to mothers, and their understanding was not evaluated. 

Investigation also revealed that mini syringe drivers used for infusion were shared between patients and did not undergo adequate disinfection between patient uses. The hospital cleaning staff were interviewed, and their cleaning methods were observed. It was found that syringe drivers were not consistently cleaned and disinfected between patients. There was no written standardized cleaning protocol for the cleaning staff to adhere to. Personal protective equipment such as gowns and gloves were not changed between patients if they were not known to carry multidrug resistant organisms. 

The environmental cleaning was noted to have been inadequately performed in the NICU, and the situation was aggravated when the dilution of the disinfectant used was incorrect. The cleaning of NICU environments was performed by hospital contract cleaning staff and was found to be inadequate due to a lack of proper training and clear delineations of roles and responsibilities. It was also observed that the sinks in the patient areas were not used appropriately. Organic materials were put into the sinks. Furthermore, the sinks did not have barricades to prevent the surrounding areas from being contaminated by splashes from the sinks.

### 3.4. Strengthening of Infection Control Intervention

#### 3.4.1. Geographic Cohorting and Contact Precautions

Infants with positive CRAB cultures were cohorted to an isolation room that was separated from infants with negative culture. Nurses were also cohorted to care for either those who had CRAB or those without. Proper use of personal protective equipment (PPE) was reinforced and audited with special attention given to the practice of discarding gowns and gloves before leaving a patient’s room or cubicle. The number of visiting teams from other units or departments to the NICU was limited to three at any one time.

#### 3.4.2. Education

HCWs were educated on proper donning and doffing of PPE. Safety rounds coupled with audits and feedback were performed regularly to reinforce compliance with hand hygiene and PPE use. In addition, mothers were re-educated by nurses on proper milk handling procedures.

#### 3.4.3. Establishment of Guidelines for Handling of EBM

The positive CRAB isolated from the sink in the milk preparation room triggered the revision of milk handling practices by both nurses and mothers as well as the development and implementation of new milk-handling and storage protocols. All used breast pumps, including the mothers’ personal pumps, were advised to be sterilized, dried and assembled before storage. Nurses were retrained in milk preparation and handling. Audits and feedback from infection control link nurses were conducted frequently to ensure all the nurses understood and complied with the protocol. 

Besides the drafting of a locally contextualized milk-handling protocol, terminal cleaning was conducted in the milk-handling room. All stored milk samples in the NICU refrigerators were discarded with consent from the mothers to prevent all sorts of possible cross-contamination, followed by extensive decontamination of the refrigerators. Several interim measures were promptly adopted, and an emergency budget was consigned towards renovation of the milk-handling and storage room. The interim measures included relocating the milk-handling room to another area that contained a proper sink, and temporary barriers were erected to contain splashes and avoid contamination of surrounding surfaces during washing of used equipment. This was a temporary measure while a dedicated room was constructed. This room design consisted of two separate areas, a dirty utility area where the sink would be placed and a clean area dedicated to milk storage and preparation. 

#### 3.4.4. Environmental Cleansing and Disinfection

Intensified environmental decontamination was conducted in all NICU environments, using 1000 ppm (5.25–6%) sodium hypochlorite (Clorox^®^) solution. HCWs were re-educated on the use of correct concentrations. To achieve strict adherence, a cleaning video and checklist were developed for reference. A cleaning audit team was formed to assess the competencies and cleaning practices of cleaning staff. An audit was performed through qualitative measurements using a fluorescent gel product (Glo Germ^TM^). Detection of residual Glo Germ^TM^ after cleaning required the HCWs involved to undergo re-education on disinfection protocols. We noted that the mini syringe drivers were within close contact with patients as these were located within the incubators themselves. The mini syringe drivers were therefore specified for sterilization with terminal high-level disinfectant (Virusolve^®^+), and an urgent purchasing of new syringe drivers was overseen to reduce the sharing of these devices between infants. 

Terminal cleaning of the sink and the sink traps was also conducted. This included complete dismantling of the sink and traps, sending the residual sediments for culture, cleaning of the traps thoroughly with detergent, with a final soak in 5000–10,000 ppm Clorox^®^ solution for 30 min before re-assembling. Repeat swabs were taken to ensure clearance. To prevent the contamination of sinks, “Guidelines for Clinical Handwashing Sinks in Patient Area” and a poster was developed that clearly stated that hand-washing sinks should not be used to discard organic material/fluids or used to wash equipment. Engineers were consulted to erect barriers adjacent to the sinks and to convert them to trapless sinks wherever possible. 

## 4. Discussion

Here, we report a rapid identification of an outbreak of XDR *A. baumannii* resulting from an established hospital-wide passive MDRO surveillance system and the containment within two months after the formation and action of a multidisciplinary outbreak team. Undeniably, the persistence of *A. baumannii* in ICUs is reported worldwide currently, ascribed to their ability to survive on inanimate surfaces as well as their reduced susceptibility to biocides [14,15]. However, data from daily multidrug-resistant microorganism surveillance conducted by the infection control team in our hospital showed that such bacteria were not isolated in the NICU for the past year. This ruled out the possibility of CRAB persistence in our unit, and inter-ward transmission was the most probable cause of the onset of this outbreak. Along with this, a lapse in infection control measures had possibly further exacerbated the situation. The use of disinfectants at sub-lethal concentrations, the sharing of medical devices among neonates and the conceivable gaps observed in hand-hygiene practices were found to be contributory factors to this outbreak. 

To identify the possible sources of the pathogen, PFGE analysis was conducted on all clinical and environmental strains. The identical clone exhibited by clinical isolates and isolates found from the sink in the milk-preparation room, syringe drivers and hands of HCWs prior to handwashing indicated that these sites likely served as reservoirs. Following this, intensified sink refurbishment and decontamination were performed. All sink traps were disassembled and soaked with sodium hypochlorite solution to achieve high-level disinfection. A refurbishment of the milk room was conducted, whereby separate rooms were formed to contain sinks for different purposes. For instance, the sink used for handwashing was separated from the sink used to prepare milk to reduce splash contamination. Such intervention was found to be effective for eradicating CRAB based on other outbreaks described in the literature. La Forgia’s study demonstrated that weekly complete sink flushing with sodium hypochlorite successfully eliminated CRAB that persisted in an ICU [16]. Similarly, an infection-control team in Seoul National University Children’s Hospital in Korea ceased the transmission of CRAB in their PICU after sink replacement [17]. 

Besides environmental decontamination, a protocol for cleaning of shared mini syringe drivers was drafted, followed by the urgent purchase of more devices to prevent sharing. All HCWs in the NICU were re-educated on their hand-hygiene practices because non-compliance is often associated with transient carriage, or subsequent colonization of the pathogen, leading to its dissemination. The fact that poor hand-hygiene practice can lead to hospital outbreaks has been proven in a study in a Canadian hospital using the DebMed Electronic Hand Hygiene Compliance System. The monitoring system showed that hospital outbreaks tend to occur in units with lower hand-hygiene adherence, but the outbreaks can be controlled once there is rapid improvement in the hand-hygiene performance [18]. Moreover, hand-hygiene adherence was found to be an important intervention in controlling a silent *Klebsiella pneumoniae* outbreak that occurred in a hospital in Eastern Turkey [19]. Notably, although hand hygiene compliance among staff in the NICU had always achieved a good percentage of more than 85%, the compliance levels had further risen to above 90% and consistently close to 100% after the enhanced education.

PFGE revealed that *A. baumannii* isolated from the milk room’s sink, syringe drivers and HCWs’ hands were within the same cluster, with a similarity of 95.2%. Such findings agreed with other studies showing that most institutional outbreaks caused by *A. baumannii* belong to a single clone [10,20]. Isolates recovered from the sink, hands of nurses and syringe drivers shared similar pulsotypes with the clinical isolates, indicating that pathogen cross-transmission among neonates was due to contact with contaminated medical items and transient colonization of nurses’ hands. Such a finding is consistent with other studies showing that outbreaks of *A. baumannii* occurred via the contaminated hands of HCWs after contacting colonized patients or contaminated environmental sites [21]. Similar antibiograms exhibited by these strains further confirmed their close relatedness. Unlike clinical isolates, an isolate of *A. baumannii* (E4) from NICU environments showed diverse genotypes with different antimicrobial susceptibility patterns, suggesting that this isolate was brought into our unit from different origins. It was found to be genetically different from those outbreak isolates and was not multidrug resistant. It was reported that *A. baumannii* can colonize healthy human skin flora and form biofilms without causing any invasion [15]. By working in the NICU, this skin colonizer sheds into the environment easily, and this explains the diverse genotype exhibited by this environmental strain. The decision to discard all expressed breast milk (EBM) located within the same refrigerator shelf was a difficult decision. However, this directive was carried out to remove possible factors that may have led to cross-contamination, and thereafter, ensuring complete elimination of the pathogen from the unit. 

In this outbreak, three babies infected with CRAB died due to concurrent medical complications, while those with CRAB colonization were discharged well. Regardless of infection or colonization status, the babies in the NICU underwent daily antimicrobial mild octenidine (Octenisan^®^) baths to reduce CRAB bioburden on their skin. Such intervention was crucial as studies have proved that daily antiseptic bathing can help reduce CRAB colonization [22]. Although this intervention is crucial, as silent carriage of CRAB among neonates can act as a reservoir, it is acknowledged that neonatal skin integrity must be cared for judiciously. Therefore, any antimicrobial cleaning of the skin must be conducted with extensive precautionary measures including taking note of skin breaks and any contraindications for exclusion. Parallel to antimicrobial bathing, it is pivotal that active surveillance culture (ASC) should be implemented for all babies enrolled into NICUs in the future to prevent the establishment of CRAB persistence in our unit. A containment program of multidrug resistant Gram-negative bacteria in a NICU in Italy further highlighted the importance of active culture surveillance in reducing the circulation of ESBL-producing *Klebsiella pneumonia* strains in their NICU settings [23]. 

There are several limitations in this outbreak investigation. Firstly, we would like to emphasize that this is a descriptive study that was carried out retrospectively, and no case-control study was performed due to the small number of colonized/ infected patients. Thus, the risk factors for CRAB acquisition or infection cannot be identified. Second, the sudden declaration of the outbreak prompted the simultaneous implementation of multi-faceted infection control measures with an aim to stop the isolation of CRAB cases immediately. Hence, the degree of effectiveness of each intervention in limiting CRAB spread cannot be evaluated. Owing to the urgent need for a quick pathogen-source identification, instead of using a whole genome sequencing technique, we used PFGE analysis to determine the genetic links among isolates. We cannot deny the absolute discriminatory power of whole genome sequencing (WGS) in determining the genetic linkage and postulating the transmission events of the isolates involved in the outbreak. However, PFGE is the most affordable and clinically relevant technique for the purpose of outbreak control. 

## 5. Conclusions

In conclusion, the first encounter of the CRAB outbreak had brought about formidable consequences to critically ill infants in the NICU. The occurrence of this outbreak is never a coincidence, but rather reflects the gaps seen within the infection control measures specialized for the NICU. Additionally, it serves as a warning for the emergence of more notorious pathogens that surpass current infection-control policies. Therefore, ongoing surveillance for MDRO, along with consistent adherence to established infection control strategies, are essential for prompt outbreak identification and termination of the chain of transmission.

## Figures and Tables

**Table 1:** Line listing of 9 neonates involved in the outbreak.

Infants	GA, Weeks	BW, g	Admission Diagnosis	Isolation Date	Specimen	Invasive Procedure	Colonization/ Infection	30 Days Mortality
1	27	845	Presumed sepsis	3 January 2017	Tracheal aspirate	Yes	Colonization	No
2	30	1305	RDS & NEC	10 January 2017	Nasopharyngeal swab	Yes	Colonization	No
3	33	1265	Omphalocele Presumed sepsis	29 January 2017	Omphalocele swab	Yes	Infection	Yes
4	33	1635	Perforated NEC	2 February 2017	Throat swab	Yes	Colonization	No
5	31	1515	RDS & NEC	29 January 2017	Blood	Yes	Infection	Yes
6	31	1535	Pneumonia	8 February 2017	Tracheal secretion	Yes	Infection	No
7	31	1730	Resolved clinical sepsis	1 February 2017	Rectal swab	Yes	Colonization	No
8	27	570	RDS	31 January 2017	Blood	Yes	Infection	Yes
9	34	1725	Resolved clinical sepsis	12 February 2017	Eye swab	Yes	Colonization	No

RDS = respiratory distress syndrome, NEC = necrotizing enterocolitis.

**Table 2:** Isolation of *A. baumannii* from various environmental sites.

Surveillance Swabs	Isolation of *A. baumannii*
26 environmental surfaces	1—Mini syringe driver 3—Sink in milk preparation room
10 HCW fingerprints	1
7 contacts screened	0

HCW = healthcare worker.

**Table 3:** Antibiograms of *Acinetobacter baumannii*.

Isolates	Antimicrobial Susceptibility Profile														
	AP	SXT	SAM	AMC	AN	CN	CXM	CAZ	CTX	CIP	IMP	MEM	TZP	CRO	CT
Patient 1	R	R	R	R	R	R	R	R	R	R	R	R	R	R	S
Patient 2	R	R	R	R	R	R	R	R	R	R	R	R	R	R	S
Patient 3	R	R	R	R	R	R	R	R	R	R	R	R	R	R	S
Patient 4	R	S	R	R	R	IN	R	R	R	R	R	R	R	R	S
Patient 5	R	R	R	R	R	R	R	R	R	R	R	R	R	R	S
Patient 6	R	R	R	R	R	R	R	R	R	R	R	R	R	R	S
Patient 7	R	R	R	R	R	R	R	R	R	R	R	R	R	R	S
Patient 8	R	R	R	R	R	R	R	R	R	R	R	R	R	R	S
Patient 9	R	R	R	R	R	R	R	R	R	R	R	R	R	R	S
Fingerprint before hand washing (E1)	R	R	R	R	R	R	R	R	R	R	R	R	R	R	S
Syringe driver (E2)	R	R	R	R	R	R	R	R	R	R	R	R	R	R	S
Sink in Milk Preparation Room (E4)	R	S	S	S	S	S	S	S	IN	S	S	S	S	IN	S
Sink in Milk Preparation Room (E3)	R	R	R	R	R	R	R	R	R	R	R	R	R	R	S

AP = ampicillin, SXT = trimethoprim-sulfamethoxazole, SAM = ampicillin-sulbactam, AMC = amoxicillin-clavulanate, AN = amikacin, CN = gentamicin, CXM = cefuroxime, CAZ = ceftazidime, CTX = cefotaxime, CIP = ciprofloxacin, IMP = imipenem, MEM = meropenem, TZP = piperacillin-tazobactam, CRO = ceftriaxone, CT = colistin.

## Data Availability

All data presented in this study are available in this published article.

## References

[1] Naing S.Y., Hordijk J., Duim B., Broens E.M., van der Graaf-van Bloois L., Rossen J.W., Robben J.H., Leendertse M., Wagenaar J.A., Zomer A.L. (2022). Genomic investigation of two Acinetobacter baumannii outbreaks in a veterinary intensive care unit in the Netherlands. Pathogens.

[2] Chaiben V., Yamada C.H., Telles J.P., de Andrade A.P., Arend L.N.V.S., Ribeiro V.S.T., Dantas L.R., Suss P.H., Tuon F.F. (2022). A carbapenem-resistant Acinetobacter baumannii outbreak associated with a polymyxin shortage during the Covid pandemic: An in vitro and biofilm analysis of synergy between meropenem, gentamicin and sulbactam. J. Antimicrob. Chemother..

[3] Risser C., Pottecher J., Launoy A., Ursenbach A., Belotti L., Boyer P., Willemain R., Lavigne T., Deboscker S. (2022). Management of a major carbapenem-resistant Acinetobacter baumannii outbreak in a French intensive care unit while maintaining its capacity unaltered. Microorganisms.

[4] Alrahmany D., Omar A.F., Harb G., El Nekidy W.S., Ghazi I.M. (2021). Acinetobacter baumannii infections in hospitalized patients, treatment outcomes. Antibiotics.

[5] Yap P.S.X., Ahmad Kamar A., Chong C.W., Yap I.K.S., Thong K.L., Choo Y.M., Md Yusof M.Y., Teh C.S.J. (2016). Intestinal carriage of multidrug-resistant gram-negative bacteria in preterm-infants during hospitalization in neonatal intensive care unit (NICU). Pathog. Glob. Health.

[6] Gramatniece A., Silamikelis I., Zahare I., Urtans V., Zahare I., Dimina E., Saule M., Balode A., Radovica-Spalvina I., Klovins J. (2019). Control of Acinetobacter baumannii outbreak in the neonatal intensive care unit in Latvia: Whole-genome sequencing powered investigation and closure of the ward. Antimicrob. Resist. Infect. Control.

[7] Pogue J.M., Zhou Y., Kanakamedala H., Cai B. (2022). Burden of illness in carbapenem-resistant Acinetobacter baumannii infections in US hospitals between 2014 and 2019. BMC Infect. Dis..

[8] Lv Y., Xiang Q., Jin Y.Z., Fang Y., Wu Y.J., Zeng B., Yu H., Cai H.M., Wei Q.D., Wang C. (2019). Faucet aerators as a reservoir for Carbapenem-resistant Acinetobacter baumannii: A healthcare-associated infection outbreak in a neurosurgical intensive care unit. Antimicrob. Resist. Infect. Control.

[9] Gokmen T.G., Akcimen B., Kayar B., Marzi M., Koksal F. (2016). The outbreak of Acinetobacter baumannii producing OXA-23 and OXA-51 type carbapenemases in a state hospital. J. Exp. Clin. Med..

[10] Ulu-Kilic A., Gundogdu A., Cevahir F., Kilic H., Gunes T., Alp E. (2018). An outbreak of bloodstream infection due to extensively resistant Acinetobacter baumannii among neonates. Am. J. Infect. Control.

[11] Gottesman T., Fedorowsky R., Yerushalmi R., Lellouche J., Nutman A. (2021). An outbreak of carbapenem-resistant Acinetobacter baumannii in a COVID-19 dedicated hospital. Infect. Prev. Pract..

[12] Chen T.L., Siu L.K., Wu R.C., Shaio M.F., Huang L.Y., Fung C.P., Lee C.M., Cho W.L. (2007). Comparison of one-tube multiplex PCR, automated ribotyping and intergenic spacer (ITS) sequencing for rapid identification of Acinetobacter baumannii. Clin. Microbiol. Infect..

[13] Seifert H., Dolzani L., Bressan R., van der Reijden T., van Strijen B., Stefanik D., Heersma H., Dijkshoorn L. (2005). Standardization and interlaboratory reproducibility assessment of pulsed-field gel electrophoresis-generated fingerprints of Acinetobacter baumannii. J. Clin. Microbiol..

[14] Babaei M.R., Sulong A., Hamat R.A., Nordin S.A., Neela V.K. (2015). Extremely high prevalence of antiseptic resistant Quaternary Ammonium Compound E gene among clinical isolates of multiple drug resistant Acinetobacter baumannii in Malaysia. Ann. Clin. Microbiol. Antimicrob..

[15] Sarshar M., Behzadi P., Scribano D., Palamara A.T., Ambrosi C. (2021). Acinetobacter baumannii: An ancient commensal with weapons of a pathogen. Pathogens.

[16] La Forgia C., Franke J., Hacek D.M., Thomson Jr R.B., Robicsek A., Peterson L.R. (2010). Management of a multidrug-resistant Acinetobacter baumannii outbreak in an intensive care unit using novel environmental disinfection: A 38-month report. Am. J. Infect. Control.

[17] Landelle C., Legrand P., Lesprit P., Cizeau F., Ducellier D., Gouot C., Bréhaut P., Soing-Altrach S., Girou E., Brun-Buisson C. (2013). Protracted outbreak of multidrug-resistant Acinetobacter baumannii after intercontinental transfer of colonized patients. Infect. Control Hosp. Epidemiol..

[18] Kovacs-Litman A., Muller M.P., Powis J.E., Ricciuto D., McGeer A., Williams V., Kiss A., Leis J.A. (2021). Association between hospital outbreaks and hand hygiene: Insights from electronic monitoring. Clin. Infect. Dis..

[19] Duman Y., Ersoy Y., Gursoy N.C., Toplu S.A., Otlu B. (2020). A silent outbreak due to Klebsiella pneumoniae that co-produced NDM-1 and OXA-48 carbapenemases, and infection control measures. Iran. J. Basic Med. Sci..

[20] Maciel W., da Silva K., Croda J., Cayô R., Ramos A., de Sales R., de Souza G.d.A., Bampi J.B., Limiere L., Casagrande J. (2018). Clonal spread of carbapenem-resistant Acinetobacter baumannii in a neonatal intensive care unit. J. Hosp. Infect..

[21] Wang C.-H., Li J.-F., Huang L.-Y., Lin F.-M., Yang Y.-S., Siu L.K., Chang F.-Y., Lin J.-C. (2017). Outbreak of imipenem-resistant Acinetobacter baumannii in different wards at a regional hospital related to untrained bedside caregivers. Am. J. Infect. Control.

[22] Fan C.-Y., Lee W.-T., Hsu T.-C., Lee C.-H., Wang S.-P., Chen W.-S., Huang C.-H., Lee C.-C. (2019). Effect of chlorhexidine bathing on colonization or infection with Acinetobacter baumannii: A systematic review and meta-analysis. J. Hosp. Infect..

[23] Saporito L., Graziano G., Mescolo F., Amodio E., Insinga V., Rinaudo G., Aleo A., Bonura C., Vitaliti M., Corsello G. (2021). Efficacy of a coordinated strategy for containment of multidrug-resistant Gram-negative bacteria carriage in a Neonatal Intensive Care Unit in the context of an active surveillance program. Antimicrob. Resist. Infect. Control.

